# Supplementary material for: A Rapid and High Throughput MIC Determination Method to Screen Uranium Resistant Microorganisms
Source: Methods Protoc. 2020 Mar 3;3(1):21. doi: 10.3390/mps3010021 (PMC7189662; doi:10.3390/mps3010021)
Supplement: Supplementary file 1 [file mps-03-00021-s001.pdf]

methods and protocols

MDPI

Article

A Rapid and High Throughput MIC Determination Method to Screen Uranium Resistant Microorganisms

Meenakshi Agarwal *, Rajesh Singh Rathore and Ashvini Chauhan

Environmental Biotechnology Laboratory, School of the Environment, 1515 S. Martin Luther King Jr. Blvd., FSH Science Research Center, Florida A&M University, Tallahassee, FL 32307, USA; rajeshsingh1.rathore@famu.edu (R.S.R.); ashvini.chauhan@famu.edu (A.C.)

* Correspondence: meenakshi.agarwal@famu.edu; Tel.: +1-850-405-8900

Received: 27 January 2020; Accepted: 28 February 2020; Published: 3 March 2020

Table S1. MIC levels of different microbial isolates, along with their susceptible concentration values against uranium.

Type of Microorganism	Name	Identification by 16S or 18S Gene Sequencing	MIC Value	Susceptibility Value
**Bacteria**	SRS-11-W-2017	*Pseudomonas* sp.	6 mM	4 mM
	SRS-2-W-2017	*Serratia marcescens*	7 mM	7 mM
	SRS-19-S-2018	*Lysinibacillus* sp.	6 mM	1 mM
	MA-5-S-2018	*Stenotrophomonas* sp.	7 mM	6 mM
	SRS-9-S-2018	*Serratia* sp.	7 mM	7 mM
	SRS-146-S-2018	*Burkholderia* sp.	8 mM	6 mM
	SRS-104-S-2018	*Bacillus megaterium*	5 mM	4 mM
	SRS-190-W-2019	*Bradyrhizobium* sp.	3 mM	2 mM
	SRS-88-S-2018	*Pseudomonas vancouverensis*	5 mM	4 mM
	SRS-115	*Paenibacillus* sp.	2 mM	2 mM
**Yeast or yeast-like fungi**	SRS-4-S-2018	*Aureobasidium* sp.	5 mM	1 mM
	MA 1694	*S. cerevisiae*	6 mM	6 mM
	SRS-45-S-2018	*Rhodotorula mucilaginosa*	6 mM	6 mM
**Fungi**	SRS-40-S-2018	*Penicillium* sp.	20 mM	16 mM
	SRS-64-S-2018	*Penicillium* sp.	20 mM	10 mM
	SRS-17-S-2019	*Fusarium oxysporum*	10 mM	2 mM
	SRS-21-S-2019	*Aspergillus* sp.	8 mM	2 mM

© 2020 by the authors. Licensee MDPI, Basel, Switzerland. This article is an open access article distributed under the terms and conditions of the Creative Commons Attribution (CC BY) license (http://creativecommons.org/licenses/by/4.0/).
